# Contribution of the Alive & Thrive–UNICEF advocacy efforts to improve infant and young child feeding policies in Southeast Asia

**DOI:** 10.1111/mcn.12683

**Published:** 2019-02-22

**Authors:** Isabelle Michaud‐Létourneau, Marion Gayard, David Louis Pelletier

**Affiliations:** ^1^ Department of Social and Preventive Medicine, School of Public Health Université de Montréal Montreal Quebec Canada; ^2^ Department of Family Medicine and Emergency Medicine Université de Sherbrooke Longueuil Quebec Canada; ^3^ Division of Nutritional Sciences Cornell University Ithaca New York USA

**Keywords:** advocacy, contribution analysis, contribution story, developmental evaluation, policy change, theory of change

## Abstract

Evaluating the impact of advocacy for policy change presents many challenges. Recent advances in the field of evaluation, such as contribution analysis (CA), offer guidance on how to make credible claims regarding such impact. The purposes of this article are (a) to detail the application of CA to assess the contribution of an advocacy initiative to improve infant and young child feeding policies and (b) to present the emergent theory of change and contribution story of how progress was achieved. An evaluation applying developmental evaluation and CA was conducted on the Alive & Thrive (A&T)–UNICEF initiative in seven Southeast Asian countries to document the extent to which policy objectives were achieved and identify key drivers of policy change. A contribution story was developed based on these experiences. The advocacy approach, which involved a four‐part process, contributed directly to (a) set the agenda of various actors and (b) create a strategic group; and indirectly to (a) set and maintain the issue on the agenda at all stages of the policy cycle, (b) support the government to carry out a set of critical tasks, and (c) extend commitment. All of this helped to achieve progress towards policy change. External influences were at play. The flexibility of A&T allowed key actors to utilize the positive external influences and address some of the negative ones through developing responsive strategies mitigating their effects. The emerging contribution story supports that A&T–UNICEF initiative contributed to the progress achieved in the participating countries.

**Key messages**

- Global guidance to improve infant and young child feeding policies exist, but advocacy efforts are needed to help countries align with international policy frameworks.
- Evaluating the impact of advocacy initiatives for policy change presents numerous challenges.
- Contribution analysis is a six‐step approach that can help explore attribution questions in complex environments.
- Using contribution analysis with developmental evaluation presents several strengths for advocacy and policy change evaluation.
- Applying these two evaluation approaches confirmed that the advocacy efforts of A&T, UNICEF, and partners contributed to progress in Southeast Asia.

## 1 INTRODUCTION

Globally, tremendous efforts have been invested to develop guidance for countries and support them in enacting policies to improve infant and young child feeding (IYCF). The adoption of the International Code of Marketing of Breast‐Milk Substitutes (referred as the Code) in 1981 by the World Health Assembly (World Health Organization [WHO], 1981), and updated through subsequent resolutions, was a landmark to regulate the practices of industry. The Innocenti Declaration on the need to protect, promote, and support breastfeeding was key to create a global action plan and reverse declining breastfeeding rates (UNICEF, 1990). It led to the Baby‐friendly Hospital Initiative, launched in 1991 by WHO and UNICEF, to foster health systems more supportive of breastfeeding, and recently updated (World Health Organization, 2018). Although those international policy frameworks seek to help countries in creating their own legislation, their existence is insufficient to trigger policy change. Advocacy strategies are needed in order to motivate and guide efforts of various institutions and ensure that countries translate international frameworks into national measures.

Advocacy is often considered more as an art than a science, and advocates require strategic capacity to be able to influence different stakeholders located at critical points within the policy process (Gillespie, Haddad, Mannar, Menon, & Nisbett, 2013; Pelletier et al., 2013; Pelletier, Menon, Ngo, Frongillo, & Frongillo, 2011). Although there is no commonly agreed definition, advocacy is used to achieve social or policy change and implies framing the issue, developing alliances, gathering and disseminating data (Reisman, Gienapp, & Stachowiak, 2007). A fast growing body of grey literature has developed to provide useful tools and guides to assess advocacy efforts (Guthrie, Louie, David, & Foster, 2005; Mansfield, 2010; Reisman et al., 2007; Tsui, Hearn, & Young, 2014). Evaluating the progress achieved thanks to policy advocacy initiatives presents numerous challenges due to: the multiple external factors that influence systems; the time frame (often requires fast adaptation to context but takes long time to produce outcomes); the myriad of actors, audiences, and interactions involved; and the fact that many tactics happen behind closed doors (Glass, 2017). Policy change is also a highly context‐sensitive phenomenon, which brings its own challenges (Riley et al., 2017). The following quote illustrates well the set of challenges faced when evaluating policy advocacy: “Evaluators must acquire and accurately weigh and synthesize imperfect information, from biaised sources with incomplete knowledge, under rapidly changing circumstances where causal links are almost impossible to establish” (Teles & Schmitt, 2011; Gardner & Brindis, 2017, p. 76). The quote also points to the problem of attribution (cause–effect), which is well recognized and highly discussed in the sources referred throughout this section.

Recent conceptual and methodological advances in the field of evaluation provide approaches that can help make credible claims about whether and how some advocacy activities contributed to policy changes. For example, contribution analysis (CA) has proved to be very useful in exploring attribution questions when conventional experimental designs cannot be carried out to assess the contribution of an intervention (Mayne, 2001, 2011, 2012). This theory‐based plausibility analysis is increasingly considered helpful in assessing the contributions of complex interventions (Dybdal & Lemire, 2010; Patton, McKegg, & Wehipeihana, 2015). CA also has been proposed for the evaluation of advocacy initiative for policy change (Gardner & Brindis, 2017). An increasing but still limited body of literature presents examples of the application of CA to various programmes and initiatives (Biggs, Farrell, Lawrence, & Johnson, 2014; Delahais & Toulemonde, 2012; Kotvojs & Shrimpton, 2007; Mayne, 2012). Several challenges in applying CA have led to a continued refinement of the approach. Although CA appears to now enter into a fourth generation (Budhwani & McDavid, 2017), some challenges still remain in its application. The present article seeks to add to the scarce literature on the application of CA to evaluate policy advocacy initiative, by providing insights from an evaluation that took place to assess the contribution of a complex advocacy initiative.

Alive & Thrive (A&T) implemented a 9‐year initiative to improve IYCF policies and practices. A first phase of implementation in Bangladesh, Ethiopia, and Vietnam led to considerable gains in building sustainable and enabling environments for the implementation and scaling up of IYCF programmes (Hajeebhoy et al., 2013). A second phase began in 2014 in which advocacy efforts expanded to include seven Southeast Asian (SEA) countries (Cambodia, Indonesia, Lao People's Democratic Republic, Myanmar, Thailand, Vietnam, and Timor‐Leste) and two African countries (Burkina Faso and Ethiopia). The advocacy efforts focused on sharing Vietnam's policy advocacy experiences and supporting organizations in other countries to advocate for either the adoption of IYCF‐friendly policies or the implementation, enforcement, or monitoring of existing policies. The work focused on three main policy areas: the Code, maternity protection, and health system strengthening. The advocacy efforts were based on the main strategies identified in the first phase of the initiative (Alive and Thrive, 2013) that evolved into a four‐part process for policy change: (a) establish and sustain partnership, (b) develop evidence base, (c) develop messages and materials, and (d) build consensus. The A&T initiative collaborated closely with UNICEF to undertake the advocacy efforts in the seven SEA countries regarding those policy areas. A real‐time evaluation of the advocacy efforts of actors in those countries took place in 2015–2017; the application of CA to this advocacy initiative is presented in this article.

The overall objective of the evaluation was to document the extent to which policy objectives were (or were not) achieved in each country and to identify the key drivers of policy change. More specifically, this evaluation sought to better understand whether and how the activities carried out within the advocacy initiative as part of a regional effort in the seven SEA countries, resulted in policy environments that were more supportive of IYCF. Although this evaluation investigated all three policy areas, the present article focuses solely on the Code. The purposes of this article are (a) to detail the application of CA to assess the contribution of a multicountry advocacy initiative and (b) to present the emergent theory of change (ToC) and contribution story of how progress was achieved.

## 2 METHODS

This real‐time evaluation used a combination of two theory‐based evaluation approaches. First, developmental evaluation (DE) was the overall framework for engaging with actors in the field. DE supports the development of an innovation by collecting various types of data to provide feedback to stakeholders and help them adapt the innovation to the emergent and dynamic context (M. Patton, 2010). Considering that the strategies and actions carried out by A&T, UNICEF, and partners were different in each country and evolved in response to the different contexts, the use of DE was especially relevant. Second, CA allows for the assessment of whether an intervention contributed to the observed effects (Mayne, 2011). CA involves elaborating a postulated ToC of a programme or intervention and testing it, while taking into account other influencing factors. It offers a systematic way to make evidence‐based causal claims in situations that do not lend themselves to conventional experimental or statistical methods. The present article focuses on CA but draws upon the data generated through DE and other sources.

### 2.1 Developmental evaluation

The DE approach took place between May 2015 and March 2017. Participants were actors working to improve IYCF in the various countries, representing A&T, UNICEF, government ministries, research institutions, and non‐governmental organizations. These participants had been primarily selected by A&T staff because they needed to identify the main actors involved in their advocacy efforts. In addition, the main researcher proposed to consider actors from additional organizations based on the main country priorities. Both of them easily reached agreement on the main actors to interview. Additional information can be found in a companion article of this supplement (Michaud‐Létourneau, Gayard, & Pelletier, 2019).

Several data collection methods were used. First, participant observation was done with five A&T staff and representatives (focal point for the various countries). This was possible because the researcher (IML) travelled with the A&T focal points who took advantage of the trip to follow‐up on the execution of activities described in the country work plans. The observation of those activities helped gain contextual knowledge and document the various ideas and actions undertaken by different actors. The researcher filled out a template identifying various items for each country: types of activities; objectives; participants; inputs, tactics, or strategies; target audience; progress; and comments or questions. At the end of each trip, the researcher and the focal point took a time for debriefing and discussing some insights. Detailed notes were taken throughout the duration of the trip.

Second, the researcher and the A&T focal point held key informant meetings with a total of 98 actors in the seven countries (Cambodia, *n* = 11; Indonesia, *n* = 19; Lao PDR, *n* = 13; Myanmar, *n* = 17; Thailand, *n* = 9; Timor‐Leste, *n* = 13; and Vietnam, *n* = 16). Considering that the status of the activities was different for each country, the discussions with the actors varied. The objectives of the key informant meetings in country were two‐fold: (a) for the researcher to introduce the real‐time evaluation and identify several actors who could be interviewed at different times throughout the evaluation period and (b) for A&T focal point to follow‐up on the execution of activities and create advocacy strategies with the country actors. A total of 13 meetings were tape‐recorded during the country visits. Tape‐recording was possible when the actors and A&T staff had already developed strong relationship and when both the researcher and the A&T focal point felt that it was appropriate to tape‐record. Although not all meetings were tape‐recorded, the researcher took detailed notes during and after those meetings. After each meeting, the researcher also debriefed with the A&T focal points to ensure having captured the most important points (especially when there was translation involved).

Third, in‐depth interviews were held with 28 actors throughout the real‐time evaluation (Cambodia, *n* = 3; Indonesia, *n* = 5; Lao PDR, *n* = 4; Myanmar, *n* = 5; Thailand, *n* = 3; Timor‐Leste, *n* = 5; Vietnam, *n* = 3). DE requires engaging with key actors during the development of their innovation. Those interactions served as data collection opportunities to generate insights based on their experiences. The number of actors and interviews per country depended on several factors: intensity of the A&T work in country, actors previously met during country visits, accessibility to some of them (e.g., accessibility was more difficult with government actors who did not speak English), involvement in A&T–UNICEF strategies, availability, and other logistic considerations. A total of 44 interviews were tape‐recorded.

Finally, a desk review was conducted with a broad diversity of documents, including research and strategic documents, A&T working documents, reports, and progress updates. A large number of documents specific to the Initiative were made available in a dropbox file by A&T and could be consulted by the A&T focal points. One of the researchers had access to this file and could select the ones to include in the analysis. The documents often varied by country, but when a document was available for most countries, those were analysed in a systematic way (e.g., the legal reviews or opinion leader assessments). Other documents came from the A&T website or were shared by A&T focal points. Most documents available on the dropbox had been read in details by one researcher (IML) and helped develop a chronology of events and strategies for each country.

All tape‐recorded interviews were transcribed verbatim. Thematic content analysis (Miles & Huberman, 1994) was carried out through an iterative process in which different types of coding (Saldaña, 2012) were performed with the QSR International's NVivo 11 software. Initially, two researchers (MG and IML) coded individually with cross‐checking (double‐coding) and ongoing discussion on the codebook. Once most categories had been developed, coding proceeded individually (MG) with discussion with the research team whenever needed.

### 2.2 Contribution analysis

The contribution story of a complex intervention can be developed by carrying out iterative steps, as summarized in Annex S1 in the Online Supplemental Materials (OSM).

**Step 1: Clarification of the cause–effect issue to be addressed**

This step involves recognizing the attribution problem and determining the specific cause–effect question to be addressed. It was acknowledged that the objectives of this real‐time evaluation involved evaluating whether the advocacy efforts carried out by A&T, UNICEF, and partners contributed to IYCF policy changes. At the onset of the evaluation, underlying questions were formulated to refine the cause–effect questions, as shown in Table 1. This shed light on the Initiative‐related complexity and its implications for the evaluation.

**Table 1:** Cause–effect issue to be addressed and initiative‐related complexity

	Inputs and processes	Context	Outcomes	Linkages among the various elements
Main evaluation question	Did the advocacy efforts carried out by A&T, UNICEF, and partners in each country contribute to IYCF policy changes?			
Underlying questions	What advocacy activities, strategies, and tactics took place in each country? How were they carried out?	What conditions and factors influenced the process of policy change?	What were the policy changes? Were the policy objectives reached?	How was progress achieved? What were the key drivers of the policy changes?
Initiative‐related complexity	The national efforts involved a range of emergent (not predetermined) activities, with actors often responding to opportunities and threats.	The Initiative was carried out in seven countries representing diverse contexts.	The outcomes were assessed as progress within a policy cycle, rather than an exclusive focus on official policy changes.	Considering the long timespan required for progress within a policy cycle, intermediate outcomes were captured and acknowledged.
Implications for the evaluation	Activities needed to be tracked prospectively.	An understanding of the broad and specific policy process in each country was required. The multiple contexts altogether needed to be accounted for.	Progress depended on the country's stage at the beginning of the Initiative and on the stage reached.	The results chain involved a large number of linkages between all the elements.

Although the underlying evaluation questions were applied to the three policy areas, the CA was carried out only on one policy area (the Code) because the actors in‐country focused primarily their work on this issue.

**Steps 2 and 3: Develop the ToC and gather existing evidence**

In a typical CA, a ToC is developed prior to data collection and confronted to available information regarding an intervention. In the present evaluation, a logic model was initially developed based on the experience of Vietnam and shared with A&T stakeholders from Headquarter and the regional office located in Vietnam. A ToC was later developed based on this logic model and included a list of assumptions.

**Step 4: Assemble and assess the contribution story**

At this stage, a first contribution story can be drafted and critically assessed based on the available evidence. This step usually guides the subsequent collection of data towards the strengthening of the weakest parts of the contribution story. In the present case, data were collected through DE during almost the entire advocacy initiative. Then, the contribution story was developed around the end of the evaluation with data covering almost all aspects of the postulated ToC.

**Step 5: Seek out additional evidence**

The contribution story drew upon data generated through DE. Two coding schemes were applied and helped shaping the contribution story, as described below.

*Systematic tracking of activities and accomplishments, and documentation of contexts*: A systematic tracking of activities and a review of all available documents (desk review, trip reports, country progressions in the interim report, etc.) allowed us to develop emergent and detailed chronologies for each country, which documented the following elements: actors, strategies, events, challenges, contextual factors, and accomplishments. Regarding accomplishments, one challenge was that they were not uniform across the countries. Thus, the evaluation did not consider an official policy change as the sole outcome of interest. Instead, it identified indications of progress within the overall policy cycle, including policy formulation or development; approval; preparation for implementation; monitoring and enforcement; and evaluation, learning, and adaptation. 1The stages enumerated here come from a paper of this supplement that specifically relates to the Code (Michaud‐Létourneau et al., 2019). Although we use the term “stage,” we acknowledge that these processes are iterative, overlapping, and nonsequential. Nonetheless, this distinction highly helps to locate findings and facilitate understanding of the policy cycle. A deeper discussion goes beyond the scope of this paper, but one can further read on the decision process in Auer (2017). The documentation of the conditions within each setting helped to gather deep contextual knowledge about the environment in which progress was being made.

*Study of the implementation of the A&T advocacy approach*: The data collected throughout the evaluation were coded and specifically analysed in regard to the four parts of the A&T advocacy approach (Annex S2 in OSM) to understand how actors carried out each part. The accumulation of data collected from all countries brought a robust overview of the diverse strategies used by the actors.

**Step 6: Revise and strengthen the contribution story**

At this step, the new data gathered usually help to build a more credible contribution story. Once done, the analysis may return to Step 4 to strengthen the contribution story. After developing our contribution story, this same iterative process helped us refine the contribution claims towards the end of the evaluation.

### 2.3 Validation of emergent findings

Over the course of the evaluation, two mechanisms helped validated the emergent findings related to the advocacy approach. First, several documents were created and shared with key actors to gather their perspectives. As an example, a midterm report was produced with preliminary insights for each country on the following: context, policy objectives, and activities; strategies and accomplishments; and selected insights on the policy process, challenges, and next steps. The content of each of the country briefs had been validated by some of the actors interviewed in country. As another example, towards the end of the study, when emerging findings on the Code had been articulated, the researchers presented them to some of the most engaged policy advocates (from A&T, UNICEF, and civil society) to gather their reaction to the emerging findings. Changes were then made to integrate their comments. Second, before each interview with actors in country, documents and data available for the country were reviewed, and questions for cross‐checking with interviewees were identified, in addition to validating emergent insights.

## 3 RESULTS

### 3.1 Initial ToC

**Figure 1:**
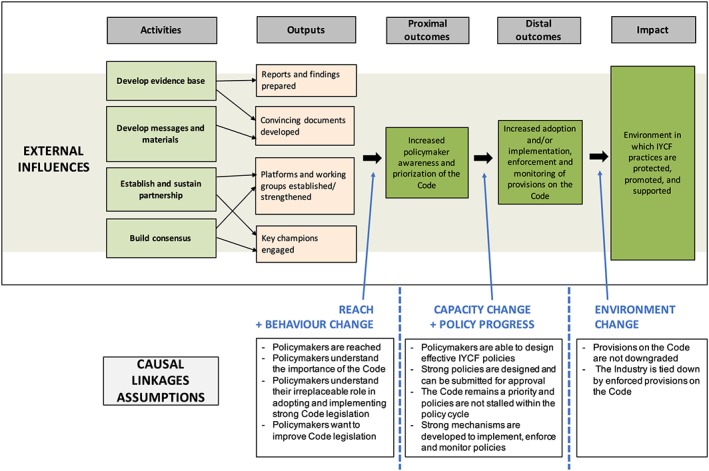
Initial ToC of the advocacy approach

In Step 2 of the CA, an initial ToC was developed from a logic model of the intervention based on the experience in Vietnam. It involved the four‐part advocacy approach used by A&T, UNICEF, and partners (Figure 1). Three linkages are illustrated from the outputs to the impact, along with a set of assumptions for each of those. Although the same advocacy approach was applied in each of the seven countries, the exact activities were not predetermined at the onset of the evaluation because, as it is usually the case for advocacy, actors respond to opportunities in each country.

### 3.2 Final contribution story

The development of the final contribution story required verifying the various assumptions related to the three main linkages identified in the ToC. The pathway by which progress was achieved is described below, with some quotes to illustrate the contributions.

#### 3.2.1 Linkage 1: Activities/outputs to proximal outcomes

The first linkage connects the activities/outputs to proximal outcomes. The assumptions for this linkage were investigated in regard to each part of the advocacy approach, and the evidence gathered is presented for each part. The first part of the advocacy approach was to build evidence. A&T initiated or supported numerous studies and analyses to develop arguments and convince actors from the government on the importance of taking action. Doing the right studies to get the right evidence at the right time was critical to advance the work, as expressed by this actor:

… when A&T came in, they looked at what they could do. They decided they would focus on operational research or sort of getting the data and information that you would need to make the advocacy that we were doing with the government much much more effective. […] They set about doing studies and information gathering to come up with a very persuasive argument as to why this policy change was necessary. […] That was the way it really worked and it was incredibly successful. UN agency representative, global, 2016.11.15

This actor referred to A&T's evidence generation in Vietnam as being highly effective, also recognizing its continuing value during phase 2 in other countries. When A&T could not directly fund studies, they sometimes facilitated the process for research to be undertaken.

A second part of the advocacy approach was to develop simplified messages and materials tailored to the target audience. This actor describes why and how it was realized:

You don't want to give this Sub‐Decree to the people especially at the national, sub‐national levels … (it) is a bit complicated for them to understand. […] That's why we actually produced the two‐pager … to make it simple and only focused on the key points, so that worked really well. […] We produced that and worked together a lot, and I'd say were impactful. […] Importantly, that two‐pager for the decision‐makers was produced on time at the National Nutrition Day last year and it was presided over by the Deputy Prime Minister, the current chairman, and also distributed widely because important, important people from the government officials were attending, so we distributed that. NGO representative, Cambodia, 2016.06.06

The packaging of findings has been mentioned several times by actors as an important strength of A&T's work. One strategy was to use multiple logos on the materials to demonstrate the closeness of the partnership and portray a consensus and commitment among the participating organizations. The availability of resources on their website was also a great asset. During country visits (and thus during participant observation), different types of actors shared positive comments on all kinds of materials. For example, in Indonesia, one government official mentioned that he explored A&T's website and had been impressed with what they had produced. In this case, a perception was that it had helped bring credibility, facilitate further collaboration, and put the work on the Code on the agenda of more actors.

A third part of the advocacy approach was to establish partnership. This part has been very effective with the creation of relationships among various actors. Further outcomes down the road were due to the contribution of two different partnerships described below.

##### The A&T–UNICEF partnership

The formal partnership between UNICEF and A&T was strong and complementary, which has without a doubt contributed to progress in the various countries. It is important to acknowledge great assets from both organizations. The long‐standing relationship between UNICEF and governments helped A&T quickly build trust and gain access to key actors, especially when A&T did not have an in‐country representation or when their work was unknown. Actors from A&T had a great understanding of the system and the resources that could be obtained from different organizations and could support well their collaborations, as described by this actor:

I mostly see positive things during this collaboration with A&T. I see that they are very supportive. It's not like they are telling us what to do and that's it and they are waiting for the results, but they are actually working with us during the process. They listen to our difficulties. Of course, one thing that I like the most is how flexible they are. […] they completely understand what's going on in the field. That's a very practical thing, I believe. Researcher in an academic institution, 2016.06.10

Actors from government and development organizations interviewed during this real‐time evaluation applauded the way by which those two organizations worked together and their complementary strengths.

##### A strategic group of actors

**Figure 2:**
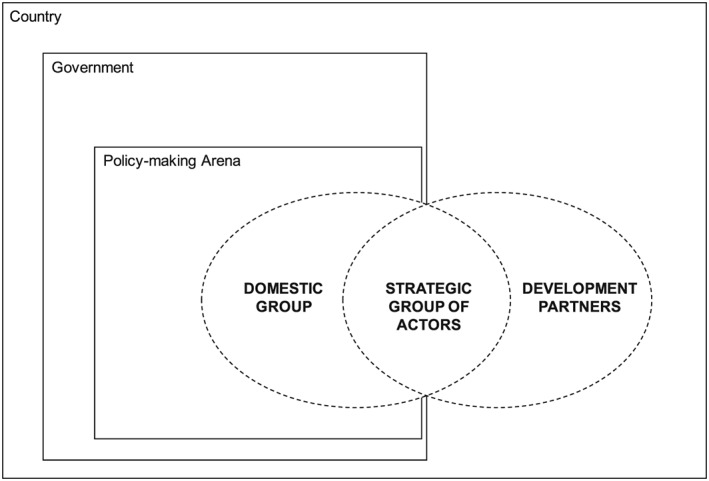
Groups of actors involved in the work on the Code

Another key aspect of the partnerships and major contribution of the advocacy approach to progress was through the creation of a strategic group of actors. In a given country, various groups were already at play. First, a domestic group was composed primarily of local actors who represented different government entities and were often directly involved with the law‐making process. An actor from one government mentioned an asset of this group, which he was part of: “I know the business process and I know which policies need what machinery and what machinery can process what policies.” Second, development partners in‐country represented another group engaged in the work. Early on, A&T used different tools to identify the right actors and organizations to engage within the different countries, which helped to foster strategic alliances. The alliances among actors from those two types of groups, fostered using the advocacy approach, led to the creation of a very effective strategic group of actors to move the work forward. All those groups had fluid boundaries and people moved in and out. Figure 2 illustrates how intertwined those groups are and their location in regard to the policy‐making arena. The strategic group created a bridge from external actors to the government to inform the policy‐making process in a positive manner. This was effective because typically, development partners are not that directly involved in the policy‐making arena of a given country.

Despite the buy‐in from several government actors from the domestic group who had been convinced of the importance of the Code, the strategic group itself had to use the advocacy approach to put the Code on the agenda of other members of government, including high‐level officials. That led to a fourth part of the advocacy approach: build consensus. Consensus building was an ongoing process pursued through a variety of strategies. The powerful influence of evidence, strategic arguments, and large, highly visible events that A&T and UNICEF helped to organize was well illustrated during the launch of The Lancet Series on Breastfeeding, which took place in several countries. In Indonesia, about 500 people attended the event, which triggered many follow‐up actions. One participant, a government representative, gave his impression on this event:

To be honest, anyone who looks at The Lancet or who has exposure to this kind of discussion would never be in a position to say that “this is something that's impossible or challenging, or that this is something that does not make sense.” This is something that's needed. […] The idea of having to implement these laws is to ensure that you get the best out of the nutrition investment, which is a long‐term economic investment over the little amount of time. Because here's what was presented: Every $1 dollar or every $1,000 dollars the industry spends to improve the marketability of their products, government cannot compete, not even below $1 dollar. It might be better for the government to take a stance of, “We do not want to compete. We just want to curtail and limit what you (the industry) can do.” And that's the general message. Government representative, Indonesia, 2016.11.06

The quote above is from an individual who was invited to the 2016 A&T–UNICEF regional policy workshop and who then became a champion for the cause and became a catalyst for change in his country. Large events were opportunities to raise awareness and influence many key actors in high‐level, well‐organized, and visible venues.

In conclusion, each part of the advocacy approach has contributed significantly to the specific outcomes presented above. Nonetheless, taken as a whole, one main contribution of the advocacy approach has been to put the Code on the agenda of various key stakeholders. This section has presented evidence on various contribution claims confirming the first set of assumptions. It has shown that key policy makers from the government were reached. They understood the importance of the Code and the role they could play, and some of them engaged themselves in moving the Code forward. This first linkage is depicted in detail in Annex S3 (OSM).

#### 3.2.2 Linkage 2: Proximal outcomes to distal outcomes

The second linkage connects the proximal outcomes to the distal outcomes. The assumptions examined related to capacity change and policy progress, which involved increasing the capacity of policy makers to develop effective IYCF policies and work towards putting them in place.

##### A set of critical tasks

The distal outcomes were conceptualized as progress within the policy cycle, that is, an advancement from one stage of the policy cycle to the following one. Those stages involved development of the Code; adoption; preparation for implementation; monitoring and enforcement; and evaluation, learning, and adaptation. For each of these stages, activities were identified. Those were carried out by the seven governments, supported by the strategic groups and linked to progress within the policy cycle. These activities were later termed “critical tasks”: none of them was sufficient to influence the whole policy cycle by itself, but governments carrying out those activities were more likely to make progress within the various stages of the policy cycle. Thus, the distal outcomes, represented as a unique box in Figure 1, involve the realization of the critical tasks. Although one paper of this supplement describes at length how those critical tasks helped translate the International Code into national measures (Michaud‐Létourneau et al., 2019), the present paper focuses on the contribution of A&T–UNICEF efforts to the realization of the critical tasks.

##### The engagement of key relevant actors to support the governments

One major contribution of the strategic group was to bring a network of people and a pool of resources to advance the work. The strategic group was able to engage key actors needed at various stages of the policy cycle. First, the engagement of key local actors helped maintain the Code on various agendas. Second, the engagement of international experts was used to support the governments in carrying out the critical tasks and achieve progress within the policy cycle. Third, the strategic group was able to extend commitment by engaging with actors beyond the health sector and with civil society.

Regarding specifically the policy‐making process, A&T and UNICEF played the role of bringing technical expertise on the Code at different times, for example, for drafting the various pieces of legislation or for providing solid arguments to counter the lobby from the industry during public hearings. By engaging the right actors to move the work forward within the policy cycle, A&T, often with UNICEF support, were “connecting the dots” within the countries. A&T was often able to fill gaps that no one could fill in a timely manner, bringing a significant contribution to the overall efforts to move the processes forward, as supported by this actor:

Sometimes, the windows of opportunity are very short, it's like opening and closing, and you find it out, and that's it, so it's important to have support and expertise available to be able to take advantage of these windows. […] Not only funding but flexibility … because even if funding is there, for example, in organizations like UNICEF … because you need to be able to … Okay, you went to the government, talked, and asked: “it's (for) tomorrow, next week, in 3 weeks … no more than 2 months.” You need to be able to respond dynamically. It depends on where you are in the policy process, but UNICEF, it would take 4 to 6 months, just, institutionally, to say, “Okay, we do that.” … Okay, A&T seems to have the flexibility of inserting this. UN agency representative, 2016.02.04

In summary, as seen in the examination of the first linkage, the advocacy approach led to the creation of a strategic group of actors. In the second linkage, this strategic group helped set and maintain the Code on various agendas and was able to engage various key actors to facilitate the undertaking of the critical tasks and progress within the policy cycle. This led to policy makers being assisted to design effective IYCF policies and submit them for adoption. The governments were also supported to develop strong mechanisms to implement, enforce, and monitor those policies. This second linkage is illustrated in detail in Annex S3 (OSM).

#### 3.2.3 Linkage 3: Distal outcomes to impact

The third linkage connects the distal outcomes to impact. As mentioned earlier, CA relied on the data collected using the DE approach to investigate A&T–UNICEF advocacy efforts. This collection of data lasted 22 months and allowed us to gather insights on a large portion of the ToC. However, as policy changes span a long timeline, this last part of the ToC was not as fully documented as the other linkages. For this reason, it is important to consider the value of intermediate outcomes (proximal and distal) when assessing progress and contributions. It is not always possible or easy to distinguish intermediate outcomes from major outcomes (impact), and those are also often intertwined among themselves (moving from an intermediate outcome to a major one), as illustrated in Table 2. This table, which only presents a few of the examples documented in the study, testifies to the progress achieved in the policy environment of the various countries. Despite that these linkages are further along in the results chain, and that the table is not very detailed, the contribution of A&T, UNICEF, and partners to reach those outcomes is well illustrated.

**Table 2:** Illustrative intermediate and major outcomes by country (All were direct or indirect result of the support from A&T, UNICEF, and partners to governments)

Countries	Intermediate outcomes	Major outcomes
Cambodia	December 2015 Approval of TOR for the oversight board, the executive working group, and the control committee. Approval of Guidelines for the implementation, monitoring and enforcement of Sub‐Decree 133 and Joint Prakas 061. April–May 2016 The government and partners developed four checklists (one per line ministry). A 1‐week workshop was carried out to get consensus on the content.	August 2014 Creation of an oversight board that led to the creation of a proper mechanism to ensure enforcement. A focal point from each line ministry was assigned to it. Creation of the control committee and the executive working group. May 2016 Consensus on the checklists for the monitoring of the Code Preparation for the monitoring of the Code
Indonesia	March 2016 The launch of the Lancet Series on Breastfeeding and the presentation of the “Cost of Not Breastfeeding” took place during the National Nutrition Day—identification of high‐level champions. May 2016 MOH attended the meeting for WHA Resolution 69.9 and the Government of Indonesia has endorsed this resolution. January 2017 Five health and nutrition organizations launched a joint statement regarding WHA Resolution 69.9.	August 2016 World Breastfeeding Week during which the Director General mentioned in front of a large forum that the Government of Indonesia supported the WHA Resolution 69.9. December 2016 The Food Standardization Unit (under the BPOM) was working on the revised draft of the Code.
Lao PDR	November 2014 Inter‐Parliamentary Union event held in Vientiane: One of the priority actions was the BMS Code. July–October 2016 MOH has organized two meetings with key stakeholders (UNICEF, SC, and other line ministries to talk about the Code). A draft of TOR for a Task Force for the Code is developed. January 2017 First meeting of the Task Force took place in which a first draft of the revised Code is discussed.	November 2014 High‐level commitment to strengthen the Code January 2017 Draft of a Prime Minister's decree for the Code
Myanmar	December 2015 The government disseminated the Order (their Code) to the formula companies in a workshop. March 2016 SC and SUN‐CSA monitor a database of BMS Code violations (through KoboCollect) and submit routine reports to government. May 2016 Second official meeting of the TWG took place. A deadline for “voluntary recall” of products violating the BMS Code was set. Monitoring reports begin to be produced and sent to the TWG.	2014 A National Order of Marketing of Formulated Food for Infant and Young Child was approved under the National Food Law. November 2015 A National TWG was established as the official national government body charged with the design and oversight of the overall process for monitoring and enforcing the National Order. 2016 Official deadline for “voluntary recall” of products violating the Order (Code): 24 July 2016. Revision of the deadline to November 2016.
Thailand	August 2015 The revised draft circulated between different ministries for comments before going to the Cabinet. January 2016 The Global Nutrition Report was launched in Bangkok, showing that Thailand was off course on all the WHA nutrition indicators. Surprised by those, the MOH committed to take action.	December 2015 The State Council has approved the draft of the BMS Code as a law (with the ban for up to 24 months). 2016 Commitment of the MOH to improve the breastfeeding rates 2017 BMS Code Act passed with more than 90% vote
Timor‐Leste	April 2016 Regional Policy Workshop in Bangkok: The BMS Code was raised as a priority among the country team members who attended.	None captured
Vietnam	December 2014 Three national dissemination workshops took place to train and inform everyone on the content of the Decree 100. September 2016 The Ministry of Planning and Investment proposed a revision to Article 7.4 in the 2012 Advertisement Law to narrow the ban on advertisement of BMS for children from 24 to 12 months of age. The Ministry of Planning and Investment proposed using the fast‐track method to approve these revisions, allowing less time for opposing viewpoints to be presented before a decision was made. The strategic actors in Vietnam developed various strategies to respond to this threat on the Code.	December 2014 Decree 100/2014/ND‐CP on marketing and use of feeding products for young children, feeding bottles, teats, and pacifiers was approved. The decree further specifies the Advertisement Law. 2015 Trainings were carried out countrywide for the monitoring of the Code. October 2016 The government (economic committee) decided to not consider the proposal to revise the Advertisement Law. The law remained unchanged, and the Code has not been downgraded.

*Note*. A&T: Alive & Thrive; BMS: Breastmilk substitutes; CSA: Civil Society Alliance; ICDC: International Code Documentation Centre; MOH: Ministry of Health; PDR: People's Democratic Republic; SC: Save the Children; SUN: Scaling Up Nutrition; TOR: Terms of reference; TWG: Technical Working Group; UNICEF: United Nations Children's Fund; WHA: World Health Assembly; WHO: World Health Organization.

#### 3.2.4 External influences

**Figure 3:**
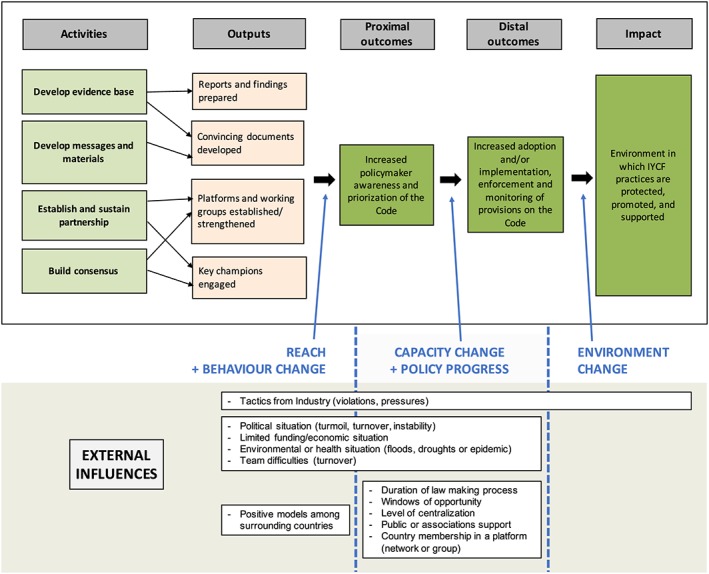
External influences affecting the advocacy efforts along the impact pathway

The creation of a credible contribution story requires attention to the potential influence of contextual factors. According to the latest thinking on contextual factors, Mayne who articulated CA, uses the term external influences and define it as the “social and economic trends at work, specific events that have occurred, other interventions overlapping with the intervention in question (with similar aims) or environmental factors at work” (Mayne, 2018, p. 5). In A&T–UNICEF advocacy initiative, several external influences appeared to have hindered or facilitated the advocacy efforts. These influences affected the work at various times, and it was possible to link them to the different set of assumptions. Figure 3 presents an aggregate list of these external influences across the seven countries and presents them according to the linkages of the ToC.

As mentioned earlier, an important feature of advocacy‐related initiatives is that many of the strategies employed by the actors are not predetermined and emerge depending on the context (Gardner & Brindis, 2017). The A&T–UNICEF initiative is no exception. The strategic group of actors had a strong capacity to analyse the contexts and were able to develop their strategies accordingly, guided by the advocacy approach. Thus, when contextual factors affected the advocacy efforts, the actors were also able to adapt to those external influences. They took advantage of the positive external influences and tried to mitigate the negative ones. Table 3 presents the implications of the external influences for the advocacy efforts and illustrates how the actors from the strategic group adapted their efforts. Because the various actors took those external influences into consideration and acted upon them, it is difficult to examine their respective level of influence on the generation of outcomes. Notwithstanding, one external influence stood out from the others: the tactics deployed by the industry. This external influence was found in all countries under study and at all time of the advocacy efforts, and represented one of the primary barriers to progress on the Code. More information can be found on those tactics in the companion article (Michaud‐Létourneau et al., 2019).

**Table 3:** External influences at play

External influences	Implications for advocacy efforts	Responsive strategies of actors	Countries
Political situation (turmoil, turnover, and instability)	The new government actors needed to be convinced; the work has to be redone. Already convinced actors did not have enough time to proceed.		Myanmar, Cambodia, Thailand, and Timor‐Leste
Limited funding/economic situation	Nutrition was not a priority anymore. Important actions/research cannot be afforded. Tools could not be put in place.	Actors built on comparative advantage and found partners with common interests and funding.	Timor‐Leste, Vietnam, Thailand, and Lao PDR
Environmental or health situation (floods, droughts, or epidemic)	Focused the attention of government and others to the emergency situation.	Actors focused their attention on getting ready for when the situation would be resolved or for an opening.	Myanmar and Lao PDR
Team difficulties (turnover)	Follow‐up on actions were more complicated.		Timor‐Leste
Tactics from the Industry (violations and pressures)	Counteracted efforts of actors (downgraded). Brought new difficulties (new form of marketing).	Actors worked to create a sentinel force to keep an eye on the internal process (e.g., using communication technology).	All countries
Positive models among surrounding countries	Actions taken by surrounding countries motivated actors to go in the same direction.	Actors used the positive model to showcase success and convince others.	Lao PDR
Duration of law‐making process	Challenges accumulated during this time (more people to convince, more risks of losing momentum, and more risks of industry attacks).		Thailand
Windows of opportunity	The timing became good for the adoption of a new regulation.	Actors tried to anticipate some of them (e.g., by staying informed on the legal landscape).	Vietnam, Lao PDR
Level of centralization	Affected the transmission of information and the implementation of the Code.	Actors strategized around the engagement of actors at different levels.	Indonesia
Lack of public or associations support	Stopped the Code during adoption.		Thailand, Indonesia
Country membership in a platform (network or group)	Created partnerships among actors. Created a communication channel among actors.	Actors used the platform to bring awareness about certain practices (e.g., conflict of interest of the Industry)	Myanmar

## 4 DISCUSSION

The use of CA helped demonstrate the contribution of the advocacy efforts carried out by A&T, UNICEF, and partners to progress regarding IYCF policies in the participating countries and revealed how progress was achieved. As proposed by Befani and Mayne (2014): “if one can verify or confirm a ToC with empirical evidence—that is, verify that the steps and assumptions in the intervention ToC were realised in practice, and account for other major influencing factors—then it is reasonable to conclude that the intervention in question has made a difference, i.e. was a contributory cause for the outcome.” Applying the main steps of CA described by Mayne allowed us to verify the assumptions of the postulated ToC and to take into account the external influences. Thus, a plausible contribution story was developed and supports that A&T–UNICEF advocacy efforts with partners made a difference and contributed to the progress achieved in the various countries. The CA also revealed how progress was achieved. The four parts of the advocacy approach helped set the agenda of various actors and allowed the creation of a strategic group, which represented a main driver of progress. Therefore, this advocacy approach was very effective, and public advocates trying to inform IYCF policies can find more information for using it (Alive and Thrive, 2016). The contribution story also uncovered the support brought by the strategic group for the realization of a set of critical tasks, previously identified as a main driver of progress within the policy cycle (see (Michaud‐Létourneau et al., 2019)). The use of the advocacy approach by the strategic group of actors also ensured setting the agenda for the Code and maintaining it there at all stages of the policy cycle. All of this was crucial to achieve progress towards policy change.

Applying CA to this initiative also brought several insights: (a) it added to an increasing but still limited body of knowledge on the application of the CA approach and (b) it demonstrated the value of CA, in combination with DE, for advocacy for policy change (APC) evaluation.

### 4.1 Insights for the application of CA

First, CA was coupled with DE, which allowed collecting data over an extended period of time and examining the postulated ToC in relation to the data collected from different sources and methods. This facilitated triangulation, which is an important aspect when undertaking CA (Budhwani & McDavid, 2017). In our case, DE facilitated triangulation through data sources and methods (M. Q. Patton, 1999), making DE a good approach to support CA.

Second, carrying out CA by drawing upon the data generated through DE allowed for the examination of most portions of the postulated ToC (up to where the countries were in the process) instead of focusing only on specific portions of the ToC. This is an important strength considering that studying only one part of the ToC, and thus taking a narrow focus, can create biases in the interpretation of the results by paying unbalanced attention to some linkages over others (Budhwani & McDavid, 2017).

Third, an innovative way was used to account for external influences. This evaluation went beyond identifying and listing external influences, by examining their respective influence on various elements of the ToC. Lemire et al. have emphasized the difficulty and the importance of accounting for the influence of specific factors and have proposed a practical framework to handle them (Lemire, Nielsen, & Dybdal, 2012). Although others have used this framework with adaptation (Biggs et al., 2014), we found it difficult to apply because of the multicountry nature of this evaluation. Moreover, although it is stated that external influences can affect results positively or negatively (Mayne, 2018), evaluators generally account only for the positive influences. In the present evaluation, the majority of external influences identified were negative and actors acted upon those by developing responsive strategies. The fact that this was an advocacy initiative, as opposed to other types of intervention, may explain this responsiveness to context. Hence, it was difficult to examine the exact degree of influence of the contextual factors on the observed results by using the framework proposed by Lemire. Instead, we studied the effects of these external influences on the advocacy efforts, by identifying the strategies of the actors responding to these influences. In addition, all the identified factors were mapped against the postulated ToC and more specifically against the different linkages in the impact pathway. In our case, it helped revealed the strong presence of industry throughout the impact pathway. The mapping of external influences and the responsive strategies of actors can help advocates of other countries working to improve national measures for the Code to anticipate the occurrence of such external influences and use similar responsive strategies.

### 4.2 Insights for APC evaluation

First, CA has been a useful framework for advocacy for policy change (APC) evaluation. This article brings a better understanding of how an advocacy approach can contribute to progress, which may have further implications for how to evaluate advocacy initiatives striving for policy change. For example, this evaluation highlights the importance of advocacy efforts to set the agenda not only at an initial stage but throughout the whole policy cycle. This is an important insight that can guide the work of advocates aiming for policy change but also of APC evaluators.

Second, combining CA with DE appears an interesting avenue for APC evaluation. In a recent book dedicated to APC evaluation, the authors suggested using the DE approach for ongoing learning and adaptation, and CA as a new analytical technique allowing to uncover the dynamics of the contribution (Gardner & Brindis, 2017). These authors referred to a survey from the Aspen/University of California San Francisco APC Evaluation on the methods most used by APC evaluators: 61% had used DE and 37% had used CA. However, those approaches are generally not used together, and we only have identified a few instances in which those were coupled (M. Q. Patton et al., 2015). Yet, as illustrated above, DE can reinforce CA. Of relevance, it is important to consider that becoming acquainted with DE and CA requires practice, time, and engagement, and allows for trials, errors, and adaptations. Funders also need to understand the need to engage with advocates and people in the field if they are to support this type of evaluation.

Regarding limitations, the fact that external influences were identified throughout the whole data collection but combined mostly at the end of the evaluation can be perceived as a limitation. However, this helped avoid biases from focusing only on a limited number of selected factors. Another potential limitation of this study may be the limited evidence gathered around the third linkage. The literature recognizes that the more we move along the impact pathway, the less evidence we are able to gather. This refers to the sphere of influence of an intervention. Indeed, moving from outputs to impact involves going from direct control (operational environment) to direct influence and eventually indirect influence (Montague, Young, & Montague, 2003).

Overall, the credibility of the contribution story is strengthened by having investigated seven countries. This is because, regardless of contexts, the detailed ToC (created based on the data collected in all countries) is seen to apply well to each country individually. The countries where the advocacy efforts were less developed also provided counter factual to reinforce the importance of some elements (the advocacy approach and the critical tasks presented in another paper of this supplement; Michaud‐Létourneau et al., 2019). Moreover, generally, drawing generalizations based on CA remains difficult because it relies on context‐specific interventions in which many external influences are at play (S. Lemire, 2010). Having compared results across several countries that used the same advocacy approach reinforce the external validity of the ToC. Therefore, the findings are likely to be generalizable for other countries working to improve national measures for the Code.

Finally, the findings presented in this paper are also aligned with the Becoming Breastfeeding Friendly Toolbox developed to guide countries in assessing their readiness to scale up breastfeeding protection, promotion, and support: This toolbox includes the Becoming Breastfeeding Friendly Index (Pérez‐Escamilla et al., 2018) based on the “Breastfeeding Gear” Model (Pérez‐Escamilla, Curry, Minhas, Taylor, & Bradley, 2012). The latter identifies eight key elements (called gears) needed for effectively scaling up breastfeeding programmes. The present study linked two of those gears: the advocacy gear and the legislation and policies gear, by illustrating that strong advocacy efforts contributed to IYCF policy improvements. Thus, the present study provides an illustration of the dynamic and interrelated nature of some of those gears. Strategic advocacy efforts that specifically target each of the seven other key gears (political will; legislation and policies; funding and resources; training and programme delivery; promotion; research and evaluation; coordination, goals and monitoring) and their underlying benchmarks (González de Cosío, Ferré, Mazariegos, Pérez‐Escamilla, & Committee, 2018) can help increase the likelihood of successfully scaling up breastfeeding programmes and foster national environments favourable to breastfeeding. Therefore, the application of the findings of this paper and its supplement goes beyond the Code.

## ETHICS APPROVAL

Ethics committees at both the University of Sherbrooke and the FHI 360 approved the research protocol.

## CONFLICTS OF INTEREST

All authors confirmed that they have no conflict of interest related to the content of this paper.

## CONTRIBUTIONS

IML designed the real‐time evaluation, played a leadership role throughout all stages of the study, collected most data, and conceptualized the paper. IML and MG conducted data analysis, interpretation of results, and drafting of the different sections of the manuscript. DLP participated in data collection, advised at all stages of the process, and collaborated in revising earlier drafts of the manuscript. All authors read, commented, and approved the final manuscript.

## FUNDING

Alive & Thrive is funded by the Bill & Melinda Gates Foundation, the governments of Canada and Ireland and is managed by FHI 360.

## Supporting information

Annex 1: Steps to carry out a contribution analysis
Annex 2: The four part of A&T advocacy approach

Annex 3
