# Supplementary material for: Contribution of the Alive & Thrive–UNICEF advocacy efforts to improve infant and young child feeding policies in Southeast Asia
Source: Matern Child Nutr. 2019 Feb 22;15(Suppl 2):e12683. doi: 10.1111/mcn.12683 (PMC6519196; doi:10.1111/mcn.12683)
Supplement: Supplementary file 1 — Annex 1: Steps to carry out a contribution analysis Annex 2: The four part of A&T advocacy approach [file MCN-15-e12683-s001.doc]

**ONLINE SUPPLEMENTAL MATERIAL**

**Annex 1: Steps to carry out a contribution analysis**

Sources : Adapted from ,

**Annex 2: The four part of A&T advocacy approach**


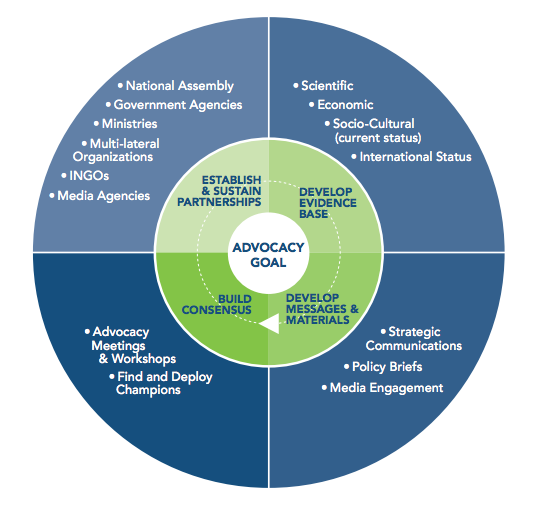


Source :
