# Supplementary figures and images for: Contribution of the Alive & Thrive–UNICEF advocacy efforts to improve infant and young child feeding policies in Southeast Asia
Source: Matern Child Nutr. 2019 Feb 22;15(Suppl 2):e12683. doi: 10.1111/mcn.12683 (PMC6519196; doi:10.1111/mcn.12683)

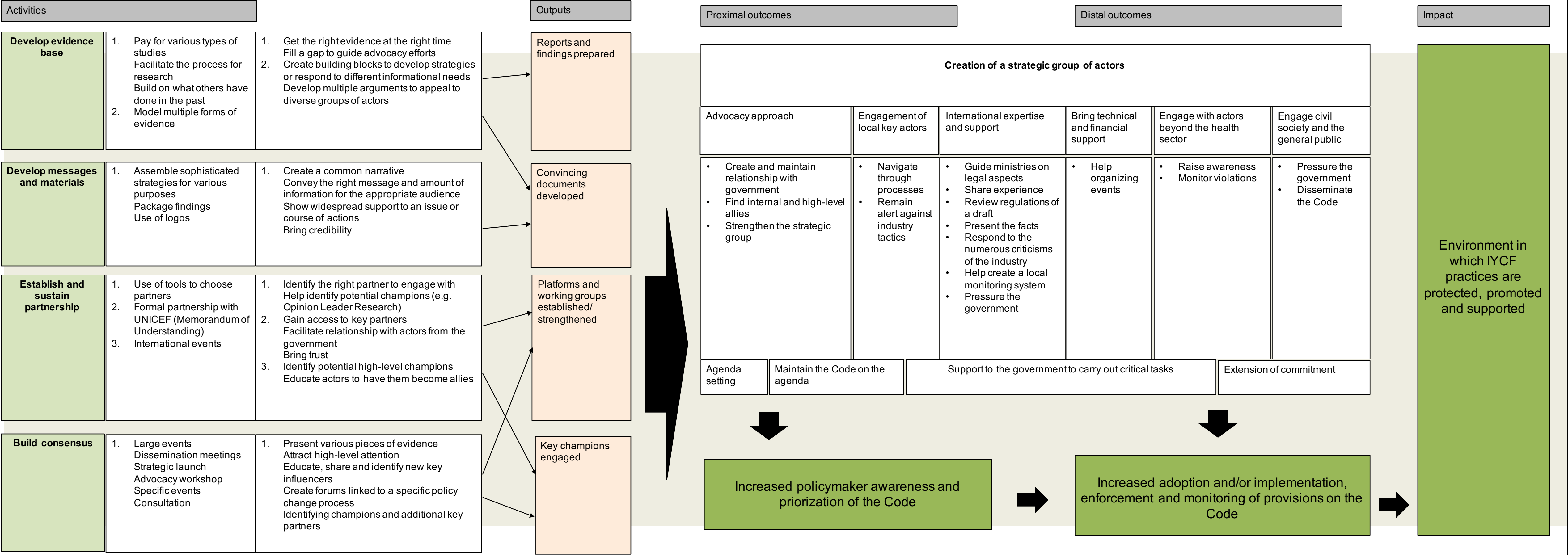

Supplement: Supplementary file 2 — Annex 3 [file MCN-15-e12683-s002.pdf]
